# Mitochondrial Ferredoxin Determines Vulnerability of Cells to Copper Excess

**DOI:** 10.1016/j.chembiol.2017.08.005

**Published:** 2017-10-19

**Authors:** Cindy Vallières, Sara L. Holland, Simon V. Avery

**Affiliations:** 1 School of Life Sciences, University of Nottingham, University Park, Nottingham NG7 2RD, UK

**Keywords:** micronutrient toxicity, Fdx2, iron-sulfur cluster, *Saccharomyces cerevisiae*, myopathy, Wilson's disease, oxidative stress

## Abstract

The essential micronutrient copper is tightly regulated in organisms, as environmental exposure or homeostasis defects can cause toxicity and neurodegenerative disease. The principal target(s) of copper toxicity have not been pinpointed, but one key effect is impaired supply of iron-sulfur (FeS) clusters to the essential protein Rli1 (ABCE1). Here, to find upstream FeS biosynthesis/delivery protein(s) responsible for this, we compared copper sensitivity of yeast-overexpressing candidate targets. Overexpression of the mitochondrial ferredoxin Yah1 produced copper hyper-resistance. ^55^Fe turnover assays revealed that FeS integrity of Yah1 was particularly vulnerable to copper among the test proteins. Furthermore, destabilization of the FeS domain of Yah1 produced copper hypersensitivity, and *YAH1* overexpression rescued Rli1 dysfunction. This copper-resistance function was conserved in the human ferredoxin, Fdx2. The data indicate that the essential mitochondrial ferredoxin is an important copper target, determining a tipping point where plentiful copper supply becomes excessive. This knowledge could help in tackling copper-related diseases.

## Highlights

• Expression level of the essential mitochondrial ferredoxin determines Cu resistance
• Copper targets the FeS domain of yeast Yah1
• Copper action at Yah1 affects downstream, essential FeS-protein function
• The function in copper resistance is conserved in the human ferredoxin, Fdx2

## Graphical Abstract

**Figure:**
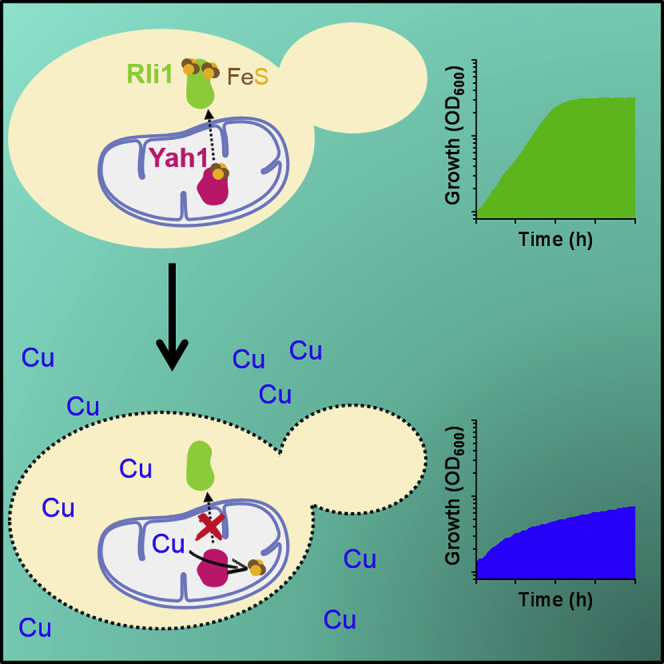

## Teaser

Vallières et al. identify the conserved ferredoxin Yah1 (Fdx2 in humans) as an important target of copper excess in cells. Copper targets the FeS domain of the ferredoxin, needed for the essential process of FeS-cluster biogenesis. This knowledge could help in tackling copper-related diseases.

## Introduction

Micronutrients that are essential for life create a dilemma for all organisms. There is a need to balance adequate supply against deleterious effects that inevitably can arise from excess (Heffern et al., 2016, Renwick, 2006). The trace element copper is essential for function of diverse enzymes such as cytochrome *c* oxidases and superoxide dismutases. Copper homeostasis is tightly regulated as Cu is highly toxic in excess. Copper homeostasis defects or elevated environmental Cu exposure can result in displacement of other essential metals from cellular constituents, inappropriate protein binding, or provocation of stress from reactive oxygen species (ROS) due to the metal's redox activity, among other reported effects (Macomber and Imlay, 2009, Pena et al., 1999). Copper toxicity has been widely described in different organisms. In humans, alterations in Cu levels or Cu-dependent functions have been associated with the pathogenesis of neurodegenerative disorders such as Wilson's disease (Bandmann et al., 2015, Kaplan and Maryon, 2016). In addition to Cu transporters and Cu-requiring enzymes, metalloproteins such as Cu-metallothioneins help to buffer free Cu in cells. However, when such defenses are inadequate, a key question remains: which molecular target(s) may be the principal “Achilles' heel” of organisms, accounting for the inhibitory action of copper? Different mechanisms of Cu toxicity have been proposed as aforementioned, but the specific target(s) remain elusive. Identification of primary cause(s) (rather than effects) of Cu action could open new opportunities for combating Cu-related disease.

Stressors and drugs commonly have essential-protein targets. These are identifiable by the essential function being sensitive to the agent in question, with knockdown of the protein producing a sensitive phenotype and overexpression conferring resistance (Avery, 2011). Iron-sulfur (FeS) clusters are protein cofactors that are among the most ROS-sensitive structures in biology (Imlay, 2006, Jang and Imlay, 2007). FeS proteins play roles in fundamental cellular processes such as the tricarboxylic acid (TCA) cycle, amino acid biosynthesis, respiratory chain, DNA synthesis and repair, mRNA translation, and FeS-protein biogenesis itself. Several FeS proteins are notoriously ROS labile, commonly those with surface exposed FeS clusters. FeS clusters are also susceptible to displacement of Fe by metals such as Cu, Ag, and Hg (Brancaccio et al., 2017, Macomber and Imlay, 2009, Tan et al., 2017, Xu and Imlay, 2012). To date, most studies of ROS- and/or Cu-sensitive FeS proteins have focused on (conditionally-) nonessential FeS proteins such as aconitase, isopropylmalate isomerase, and fumarase (Foster et al., 2014, Jang and Imlay, 2007, Jang and Imlay, 2010, Macomber and Imlay, 2009, Tan et al., 2017). Work with yeast showed that Cu stress also impairs function of the essential FeS protein Rli1 (ABCE1) (Alhebshi et al., 2012). Rli1 is highly conserved across eukaryotes and archaea, being required for ribosome biogenesis and maturation, translation initiation and termination, and ribosome recycling (Nurenberg and Tampe, 2013). Loss of Rli1 function and growth inhibition caused by Cu was due to defective FeS-cluster supply to Rli1 (after incorporation into Rli1, the FeS clusters were relatively stable) (Alhebshi et al., 2012). This indicated that a primary target accounting for inhibition lay upstream of Rli1, within the FeS-cluster synthesis or delivery pathway.

**Figure 1:**
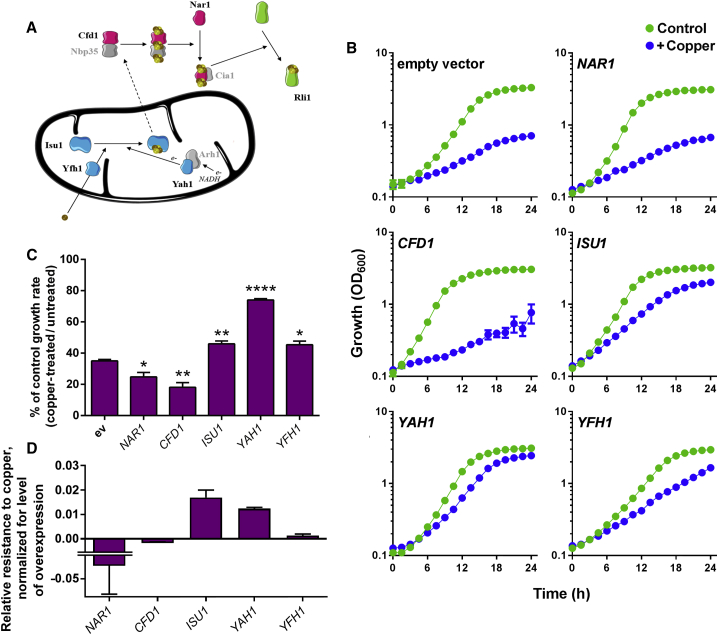
Influence of FeS Biosynthesis/Delivery Proteins on Cellular Copper Resistance (A) Simplified scheme showing FeS-cluster biogenesis and transfer to extra-mitochondrial proteins (e.g., Rli1) in the yeast model. Proteins tested in this study are shown in color. In blue are proteins of the ISC machinery: Isu1, scaffold; Yfh1, iron donor; Yah1, electron donor. In pink are proteins of the CIA machinery: Cfd1, scaffold; Nar1, cluster transfer from the scaffold complex to apoproteins. Rli1 is an essential destination protein. (B) *S. cerevisiae* BY4741 transformed with high-copy *tet* bearing plasmid, empty or overexpressing proteins of interest, were cultured in YNB medium supplemented or not with 0.6 mM Cu(NO_3_)_2_. Doxycycline was excluded to give maximal expression. Mean data are shown from triplicate independent growth experiments ± SEM. (C) Data shown are calculated from maximum growth rates (μ_max_ = ln2/g, where g is cell doubling time) determined during exponential growth, from plots presented in (B). *p < 0.05, **p < 0.01, ****p < 0.0001 according to Student's t test, two tailed. (D) Data from (C) were normalized to the relative level of overexpression for each gene (Figure S1), after subtraction of the control (ev) growth-rate effect. See also Figure S1.

In eukaryotic cells, FeS clusters are synthesized and inserted into apoproteins by the mitochondrial iron-sulfur cluster (ISC) assembly machinery and the cytosolic iron-sulfur protein assembly (CIA) machinery (Figure 1A). These pathways are well conserved from yeast to humans (Lill, 2009, Rouault, 2012, Sheftel et al., 2010). Mitochondrial ISC assembly comprises more than 15 components. *De novo* FeS-cluster synthesis on the scaffold protein Isu1 requires sulfur and iron donors, the cysteine desulfurase complex Nfs1-Isd11, and frataxin (Yfh1), respectively, as well as proteins required for electron transfer, namely the ferredoxin reductase Arh1 and the reduced ferredoxin Yah1 (Lill et al., 2014). Subsequently, the nascent cluster is transferred to mitochondrial apoproteins or exported to the cytosol. Here, maturation of cytosolic and nuclear FeS proteins such as Rli1 is supported by the CIA machinery, which includes the Cfd1-Nbp35 scaffold complex, and Nar1 and Cia1, which direct FeS clusters to apoproteins (Lill, 2009).

The lability of FeS clusters to oxygen and Cu (Chillappagari et al., 2010, Djoko and McEwan, 2013, Jang and Imlay, 2007, Macomber and Imlay, 2009) suggests that all steps in the biogenesis pathway are potentially Cu susceptible. Nevertheless, the component proteins are compartmentalized in the mitochondria and the cytoplasm, between which Cu will not be evenly distributed. Furthermore, FeS-cluster orientation in different proteins affects accessibility of Cu and ROS (Imlay, 2014, Jang and Imlay, 2007). Accordingly the FeS clusters of Rli1 itself proved Cu stable whereas cluster supply to Rli1 was not (Alhebshi et al., 2012). Those results led to the aim of this study, to characterize a primary target of Cu toxicity in cells. Here, we pinpoint the essential mitochondrial ferredoxin, Yah1, the functional ortholog of bacterial ferredoxins and human Fdx2 (Sheftel et al., 2010). The mitochondrial ferredoxin fulfilled the key criteria of a primary Cu target described above, indicating that this protein's Cu-labile FeS clusters account for downstream effects of Cu on essential FeS-protein function and on associated growth defects of cells faced with an excess of this micronutrient.

## Results

### FeS Biosynthesis/Delivery Proteins that Confer Copper Resistance

Previous work suggested that a key target of Cu toxicity may reside in the FeS-cluster biosynthesis and delivery pathway, upstream of the cytosolic FeS-recipient protein Rli1 (Alhebshi et al., 2012). A small panel of proteins representing diverse locations and functions in the pathway were analyzed here: Cfd1 and Nar1 of the CIA machinery, and Isu1, Yah1, and Yfh1 of the ISC assembly machinery. Where a protein is the major target of an agent's inhibitory action, increasing the protein's expression should confer resistance while knockdown should produce sensitivity (Avery, 2011). We also reasoned that proteins located close in a pathway to a major target would confer partial resistance if their activity can compensate some lost FeS flux through the target. We compared Cu resistance mediated by the above candidates, overexpressed through use of *tet*-promoter constructs in cells incubated without doxycycline to give maximal expression (Alhebshi et al., 2012) (Figures 1 and S1). Throughout this work, Cu was supplied at concentrations either moderately or barely inhibitory to growth of the wild-type, depending on whether the assay was for increased resistance (i.e., Figure 1) or sensitization, respectively, in the test strain(s). (*In vitro* assays of cluster turnover [below] required much lower Cu concentrations as these were with purified proteins and in simple buffers, less prone to Cu complexation [Avery et al., 1996, Macomber and Imlay, 2009].) Copper toxicity was rescued by overexpression of Isu1 and Yfh1, and particularly Yah1, of the ISC assembly machinery (Figures 1B and 1C). Isu1 and Yfh1 gave comparatively small increases in Cu resistance, but overexpression of Yah1 altered the cell doubling time from 7 hr to 3 hr at 0.6 mM Cu(NO_3_)_2_. Overexpression of the CIA pathway proteins Nar1 and Cfd1 had no protective effect. As the expression levels of each gene differed (Figure S1), we also considered the growth-rate effects after normalizing for relative degree of gene overexpression (Figure 1D). Here again, Cu resistance was highest in Isu1- and Yah1-overexpressing cells, whereas the effect of Yfh1 appeared more marginal considering its relatively high level of overexpression. The Yah1 and Isu1 proteins conferring Cu resistance are at the mitochondrial root of FeS biogenesis. The mitochondrial weakness of the BY-yeast background used here (see Discussion) has certain advantages for detecting mitochondria-targeting actions relevant to FeS biogenesis. Whereas mitochondrial genes generally are overrepresented among the Cu-sensitive set of BY-yeast deletion mutants (Bleackley et al., 2011), many individual mitochondrial proteins of course do not confer Cu resistance while many cytosolic proteins do. The latter include cytosolic Rli1 (Alhebshi et al., 2012), discussed above. Therefore, any key role for cytosolic Nar1 or Cfd1 should be similarly detectable, but our data suggested Yah1 and Isu1 as the lead candidates. We took forward Yah1 and Isu1 for further investigation, alongside Nar1 and Rli1 as cytosolic controls.

### The Essential Mitochondrial Ferredoxin Undergoes Iron Turnover in High-Copper Conditions

**Figure 2:**
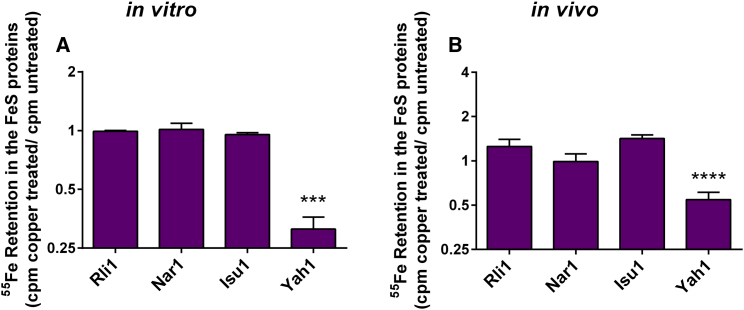
^55^Fe Turnover during Copper Treatment of Key Proteins Involved in FeS-Cluster Biosynthesis and Delivery Yeast expressing HA-tagged constructs of the specified proteins, under *tet* control from high-copy plasmids, were cultured without doxycycline to maximize expression; this overexpression gave yields of between 0.6 × 10^6^ cpm ^55^Fe (g cells)^−1^ (for Isu1-HA) and 1.6 × 10^6^ cpm ^55^Fe (g cells)^−1^ (for Yah1-HA) in immunoprecipitates of the proteins after labeling as follows (the value for the background was <0.1). (A) HA-tagged, ^55^Fe-labeled proteins were immunoprecipitated from protein extracts of cells preincubated with 180 μCi L^−1 55^FeCl_3_. Copper-resistant ^55^Fe retention by the proteins was calculated from ^55^Fe determinations after subsequent 10-min incubations with 350 μM ascorbate/100 μM histidine (Macomber and Imlay, 2009) supplemented or not with 12.5 μM Cu(NO_3_)_2_ (Cu had effect at low concentrations in these *in vitro* conditions). (B) After preloading cells with ^55^Fe (supplied at 180 μCi L^−1^, in the absence of Cu), ^55^FeCl_3_ was removed from the medium and cells incubated for 1 hr with or without 1 mM Cu(NO_3_)_2_, before immunoprecipitation of the HA-tagged proteins and ^55^Fe quantification. ***p < 0.001, ****p < 0.0001 according to Student's t test, two tailed, by comparison with data for Rli1 (which retains ^55^Fe in the Cu condition [Alhebshi et al., 2012]). All values are means from at least three independent experiments ± SEM. See also Figures S2 and S3.

We hypothesized that a primary Cu target in the FeS biogenesis/delivery pathway would have Cu-labile FeS cluster(s). We therefore compared the loss of cluster from Yah1 and Isu1 during Cu treatment by monitoring the amount of ^55^Fe in the proteins. This method provides a faithful and sensitive determination of FeS cluster in the proteins *in vitro* and *in vivo* (Pierik et al., 2009). Yfh1 was not tested, as it gave the smallest resistance to Cu (above) and radiolabeled Fe cannot be detected in the protein. We used Nar1 as a control as its overexpression did not confer Cu resistance, as well as the pathway endpoint Rli1, which has relatively stable FeS clusters (Alhebshi et al., 2012). Test proteins were expressed with hemagglutinin (HA) tags and immunoprecipitated from cells preincubated with ^55^FeCl_3_. Fe turnover from each ^55^Fe-labeled, immunoprecipitated protein was compared according to ^55^Fe release during *in vitro* incubations with a Cu(NO_3_)_2_/ascorbic acid system (Alhebshi et al., 2012, Macomber and Imlay, 2009). Results for each protein are presented as a ratio of ^55^Fe retention in the Cu versus minus-Cu conditions (data for the individual conditions are given in Figure S2). Only Yah1 showed a marked (∼80%) Cu-dependent turnover of ^55^Fe (Figure 2A). The other proteins showed some variable leakage of ^55^Fe in the absence of Cu *in vitro* (Figure S2), but the preloaded ^55^Fe only of Yah1 was susceptible to Cu addition.

We also investigated Fe turnover *in vivo*, to reflect more closely the physiological situation within cells. Cells preloaded with ^55^FeCl_3_ during culturing were subsequently incubated in ^55^FeCl_3_-free growth medium for 60 min, with or without Cu, before protein extracts were subjected to immunoprecipitation and the ^55^Fe determined in pull-downs (Figure 2B). Cu(NO_3_)_2_ was supplied at a concentration giving <10% slowing of growth rate. The mild Cu treatment did not cause a significant ^55^Fe turnover from Rli1, Nar1, or Isu1. In contrast, Cu caused a ∼50% turnover of ^55^Fe from Yah1. This decrease was not attributable to a decrease in the level of immunoprecipitated Yah1 protein (Figure S3). As reasoned earlier, mild rescue of Cu toxicity by Isu1 (Figures 1B and 1C) but absence of FeS turnover in Isu1 (Figure 2) could indicate that this protein's pathway proximity to Yah1 allows it to compensate partly for any decreased FeS flux due to Yah1 dysfunction. It is also possible that Isu1 may stabilize Yah1 from damage. As Yah1 is essential for FeS biogenesis, the Cu-dependent ∼50% loss of iron from the [2Fe-2S] cluster of Yah1 could be expected to have downstream consequences. Decreased expression of Yah1 causes decreased ^55^Fe incorporation to Rli1 (Kispal et al., 2005) and other cytosolic FeS proteins (Lange et al., 2000, Paul et al., 2015) (this would not be expected to affect the present *in vivo* assays of Cu-dependent ^55^Fe turnover with the cytosolic proteins, as the high level of Yah1 overexpression more than compensates for ∼50% Cu-dependent loss of upstream Yah1-holoprotein; Figure S1). The data suggest that a Cu-sensitive weak point in the FeS synthesis pathway may occur at Yah1.

### Yah1-Dependent Resistance to Copper

**Figure 3:**
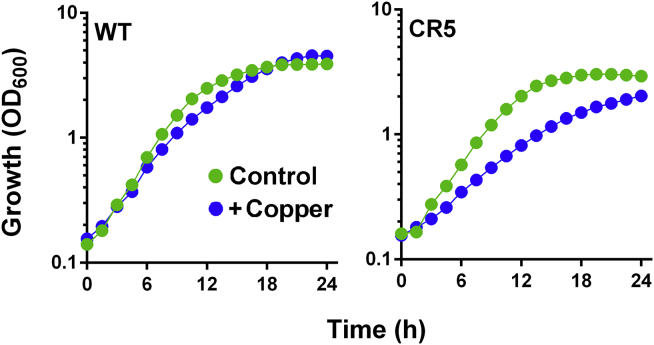
Decreased Functional Yah1 Sensitizes Cells to Copper *yah1Δ* cells expressing either wild-type (WT) *YAH1* or a ts *yah1* allele (CR5 mutant) from single-copy plasmids were cultured in YNB supplemented or not with 0.7 mM Cu(NO_3_)_2._ SEMs from triplicate independent growth experiments are smaller than the symbol dimensions. See also Figures S4 and S5.

Having established that Yah1 overexpression increases Cu resistance (Figures 1B and 1C), we tested whether decreased functional Yah1 is sufficient to produce Cu sensitivity. Absence of Yah1 (by *YAH1* deletion) causes viability loss, consistent with a putative target of toxicant action. We expressed a *yah1* allele (CR5 mutant), which has decreased Yah1-related function (Barros et al., 2002). The CR5 mutant was Cu sensitive (Figure 3). Copper exposure and genetic defects in FeS-cluster status can provoke mitochondrial Fe hyperaccumulation, including in Yah1-depleted cells (Foster et al., 2014, Lange et al., 2000). We tested Cu sensitivity in two such FeS-cluster assembly mutants with Fe hyperaccumulation phenotypes, namely *isu1Δ* (Ramazzotti et al., 2004) and *ssq1Δ* (Knight et al., 1998). Neither mutant was Cu sensitive (Figure S4), countering the possibility that Fe accumulation in Yah1-depleted cells may be the cause of their Cu sensitivity (the hint of a delayed lag-phase exit in the *ssq1Δ* mutant reflected one outlying replicate curve, not reproduced in independent replicates). As Cu is redox active and can provoke oxidative stress, we also considered whether any effect of Yah1 activity on ROS levels might be a cause of Yah1-dependent Cu resistance. However, the level of Yah1 expression did not markedly alter ROS levels measured with the ROS probe DHE, in either the absence or presence of Cu (Figure S5A).

**Figure 4:**
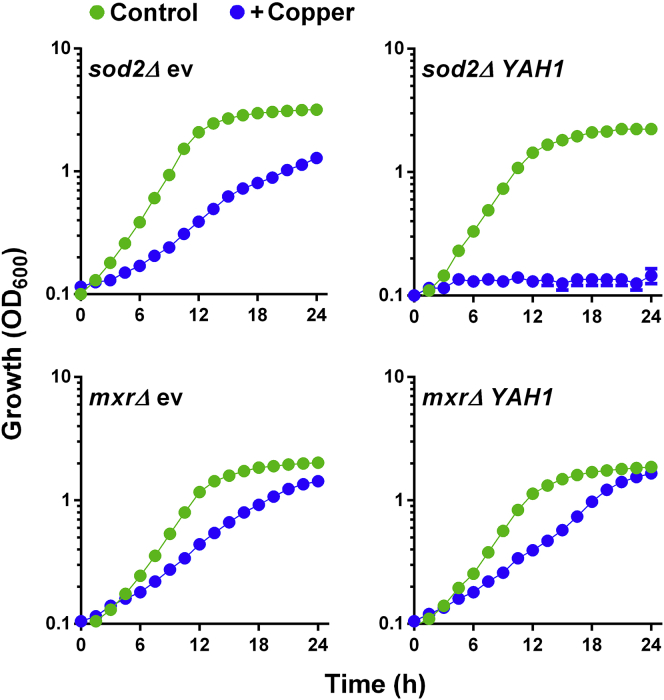
Copper Resistance with Increased Yah1 Expression Is Abolished in Strains Defective for Antioxidant Defense *S. cerevisiae mxrΔ* and *sod2Δ* mutants transformed with the *tet* bearing plasmid, either empty (ev) or overexpressing *YAH1*, were cultured in YNB medium supplemented or not with 0.6 mM Cu(NO_3_)_2_. Doxycycline was excluded to give maximal *YAH1* expression. SEMs from triplicate independent growth experiments are smaller than the dimensions of the symbols.

In contrast to the observed rescue of Cu sensitivity via overexpression of Yah1 (Figures 1B and 1C) or of pro-oxidant action via Rli1 (Alhebshi et al., 2012, Laleve et al., 2016, Vallieres and Avery, 2017), increased expression of nonessential FeS proteins is known potentially to exacerbate ROS stress. This is because of the increased pool of labile FeS which, following turnover (e.g., ROS mediated), leads to the accumulation of free Fe and further potential for ROS stress via Fe-catalyzed Fenton chemistry (Keyer and Imlay, 1996, Liochev and Fridovich, 1999). This ROS stress may be further aggravated by upregulation of Fe uptake in response to the eroding FeS status (Chillappagari et al., 2010, Foster et al., 2014, Rutherford et al., 2005). In the conditions used above (Figure 1), such potential detrimental consequences of increased Fe release from overexpressed Yah1 during Cu stress (Figure 2) appeared to be outweighed by the benefits (from Yah1 overexpression) of any rescue of essential Yah1 function. We hypothesized that this balance might be reversed under conditions more permissive for Fe-catalyzed ROS stress. Therefore, we overexpressed *YAH1* in a *sod2Δ* background, defective for scavenging of mitochondrial superoxide, which may fuel Fe-catalyzed ROS formation and further stimulate upstream FeS-cluster turnover (Imlay, 2006, Irazusta et al., 2006, Jang and Imlay, 2007). We also tested a methionine sulfoxide reductase mutant (*mxr1Δ*, *mxr2Δ* or “*mxrΔ*”) which shows impaired FeS-cluster integrity, suggested to result from elevated superoxide (Sideri et al., 2009). In these mutants, *YAH1* overexpression did not confer Cu resistance. Indeed, high Yah1 expression produced Cu hypersensitivity in *sod2Δ* cells (Figure 4). We infer that the Cu resistance that results from *YAH1* overexpression in the wild-type (Figure 1) requires lower (wild-type) levels of mitochondrial superoxide, because higher superoxide may exacerbate the toxic consequences of (Cu-dependent) Fe release from Yah1.

Yah1-dependent Cu resistance seen in the wild-type background appeared not to be related to any suppression of Fet3-dependent Fe uptake (*FET3* is upregulated in response to low FeS-cluster status [Rutherford et al., 2005]), as *FET3* expression in Cu-treated cells was not affected by Yah1 overexpression (Figure S5B). Similarly, Yah1 overexpression did not affect expression of Mrs3 or Mrs4, which mediate Fe transport across the inner mitochondrial membrane (Figure S5C). The Cu-transporting ATPase encoded by *CCC2* is the yeast ortholog of human *ATP7A* and *ATP7B*, mutations in which cause Cu homeostasis defects linked to Menkes’ and Wilson's diseases, respectively. Such defects are also associated with mitochondrial oxidative damage (Rossi et al., 2004), raising the question of whether mitochondrial ferredoxin may protect against such consequences also of internal Cu imbalance (i.e., without excess Cu addition). However, the yeast *ccc2Δ* mutant did not exhibit elevated mitochondrial ROS (Figure S5D) and is known to have a Cu-limited phenotype (Voskoboinik et al., 2001).

**Figure 5:**
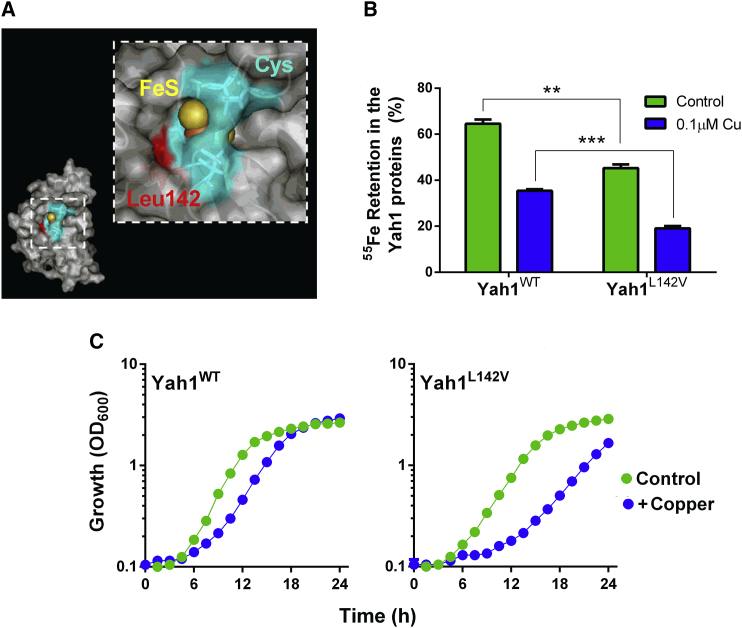
Construction and Expression of a Yah1 Mutant with Defective FeS Integrity, Conferring Copper Sensitivity (A) Location of the mutated L142V residue (in red) within the reduced yeast ferredoxin. The location of the Cys residues coordinating the FeS cluster is shaded blue. Figure prepared using the coordinates of PDB: 2MJE. (B) HA-tagged proteins were immunoprecipitated from protein extracts of cells preincubated with ^55^FeCl_3_. ^55^Fe retention by the proteins was calculated from ^55^Fe determinations before and after 10 min of incubation with 350 μM ascorbate/100 μM histidine supplemented or not with 0.1 μM Cu(NO_3_)_2_. **p < 0.01, ***p < 0.001 according to Student's t test, two tailed. All values are means from three replicate determinations ± SEM. (C) *yah1Δ* cells expressing either wild-type *YAH1* or *YAH1*^*L142V*^ from single-copy plasmids were cultured in YNB medium supplemented or not with 1 mM Cu(NO_3_)_2_. SEMs from triplicate independent experiments are smaller than the dimensions of the symbols. See also Figures S6 and S7.

The CR5 mutant described above (Figure 3) has several randomly generated mutations in Yah1, and it is not known which are responsible for the associated phenotypes (Barros et al., 2002). Hypothesizing that the FeS cluster of Yah1 is the protein's Cu-vulnerable target, we reasoned that specific mutations to further destabilize the cluster should confer Cu hypersensitivity. There are no standard approaches for manipulating FeS-cluster lability within proteins. We exploited knowledge from the cyanobacterium *Anabaena variabilis*. Its two ferredoxins have similar activities in electron transfer, but FdxH1 is relatively stable whereas FdxH2 is oxygen labile, with the residue at position 77 critical for this difference: the longer side chain of a leucine (in FdxH1) than valine (FdxH2) inhibits access of oxygen to the cavity at the FeS cluster (Singh et al., 1999, Singh et al., 2001). Based on sequence alignments, we identified a leucine (Leu-142) in the yeast mitochondrial ferredoxin (CysX_5_CysX_2_CysX_N_**Leu**XCys) corresponding to Leu-77 of FdxH1 (CysX_4_CysX_2_CysX_N_**Leu**XCys) (the Cys residues are those coordinating the FeS cluster). Leu-142 is also in the cavity leading to the FeS cluster (Figure 5A) (Webert et al., 2014). We replaced Leu-142 of Yah1 with a valine by site-directed mutagenesis, and compared *in vitro* ^55^Fe turnover in the wild-type and mutant Yah1. The Yah1^L142V^ mutant showed significantly greater loss of ^55^Fe than the wild-type protein, even in the absence of Cu (Figure 5B). (Since the assay system did not strictly exclude oxygen, the observation for the minus-Cu condition is consistent with the L142V mutation allowing increased access of oxygen to the FeS cluster [Singh et al., 2001]; see Discussion.) Yeast modified to express Yah1^L142V^ in place of wild-type Yah1 exhibited normal growth under standard conditions (Figure 5C). However, the Yah1^L142V^ mutant was Cu sensitive, with an extended lag phase and exponential-phase cell doubling time ∼1.3-fold slower than the control strain at 1 mM Cu(NO_3_)_2_ (Figure 5C). Therefore, destabilization of the Yah1 FeS-cluster produced a Cu-sensitive phenotype. The data collectively supported the hypothesis that Yah1 is a key target of Cu action.

### Rli1 Function Depends on Yah1 Expression Level during Copper Stress

**Figure 6:**
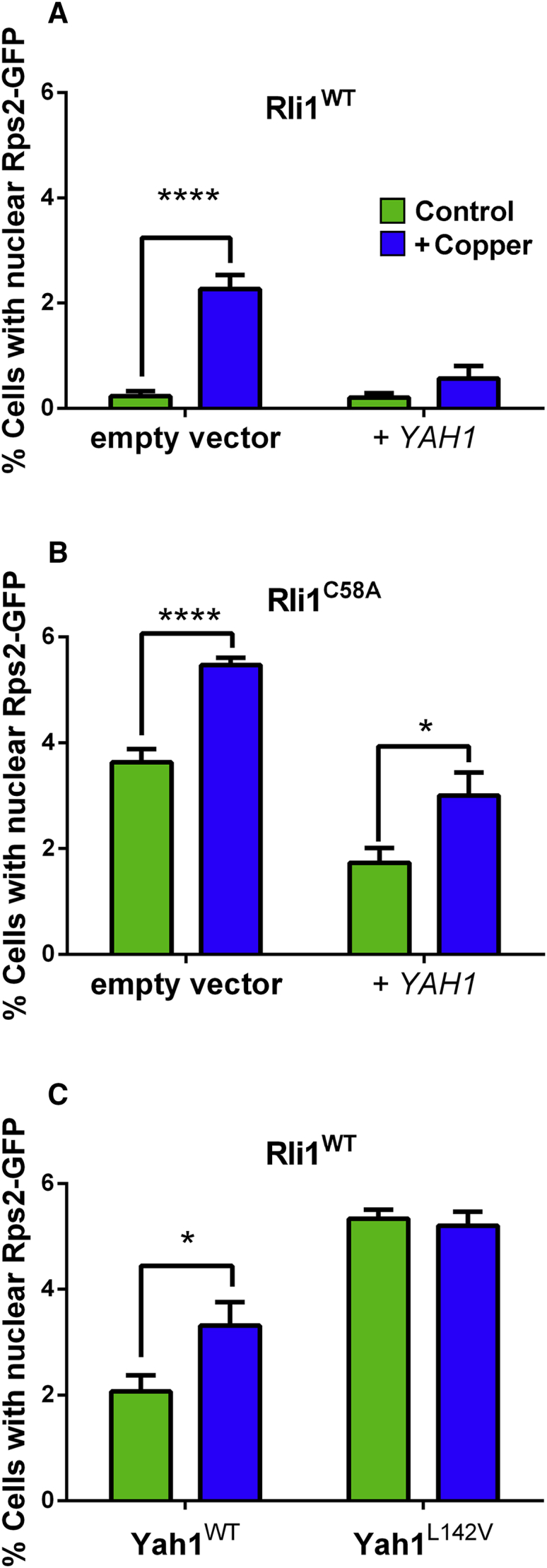
Rli1 Function Depends on Yah1 Expression Level during Copper Stress (A) *S. cerevisiae* BY4741 transformed with pRS315-*RPS2-eGFP* and a high-copy *tet* plasmid, either empty or overexpressing *YAH1* (*+YAH1*), was cultured with or without 0.35 mM Cu(NO_3_)_2_ (which affected cell doubling time <15%). Doxycycline was excluded. Cells with nuclear Rps2-GFP were enumerated after 4 hr. (B) Cells expressing Rli1^C58A^ in place of wild-type Rli1 and transformed as above were incubated for 4 hr in the absence or presence of 0.25 mM Cu(NO_3_)_2_ (this lower Cu concentration reproduced the mild <15% effect on doubling time in this Cu-sensitive mutant). (C) *yah1Δ* cells expressing either wild-type *YAH1* or *YAH1*^*L142V*^ from single-copy plasmids were incubated for 4 hr in the absence or presence of 0.35 mM Cu(NO_3_)_2_. *p < 0.05, ****p < 0.0001 according to Student's t test, two tailed. All values are means from at least three replicate determinations ± SEM, with 500 cells counted in each.

When Yah1 expression is decreased, the level of ^55^Fe incorporated to the essential cytosolic protein Rli1 is decreased (Lange et al., 2000). Therefore, perturbation of Yah1 function could explain the impaired Rli1 activity associated with Cu stress previously suggested to arise from targeting of an upstream FeS biogenesis or transfer step (Alhebshi et al., 2012). We tested the impact of Yah1 on Rli1 function during mild Cu stress using the principal *in vivo* assay for Rli1 function: nuclear export of the small ribosomal subunit Rps2. Nuclear export of fluorescence in cells expressing Rps2-GFP is a sensitive indicator of FeS-dependent Rli1 activity (Alhebshi et al., 2012, Kispal et al., 2005). As reported previously (Alhebshi et al., 2012), Cu exposure increased the proportion of wild-type cells exhibiting nuclear accumulation of Rps2-GFP, i.e., defective Rli1 function. However, *YAH1* overexpression under the same condition rescued ∼75% of nuclear Rps2-GFP export activity (Figure 6A). *YAH1* overexpression also partly rescued nuclear Rps2-GFP export in cells expressing Rli1^C58A^ (in place of the wild-type protein) (Figure 6B), a labile Rli1 construct associated with decreased export activity and Cu sensitivity (Alhebshi et al., 2012, Barthelme et al., 2007). Conversely, nuclear accumulation of Rps2-GFP was increased in the Yah1^L142V^-expressing strain (Figure 6C). This was in keeping with the FeS instability of the mutant Yah1 (Figure 5B), expected to perturb downstream FeS supply to destination proteins. In conclusion and combined with the other results, Yah1 fulfilled the anticipated criteria of a key direct protein target of Cu toxicity.

### The Human Ferredoxin, Fdx2, Confers Copper Resistance

**Figure 7:**
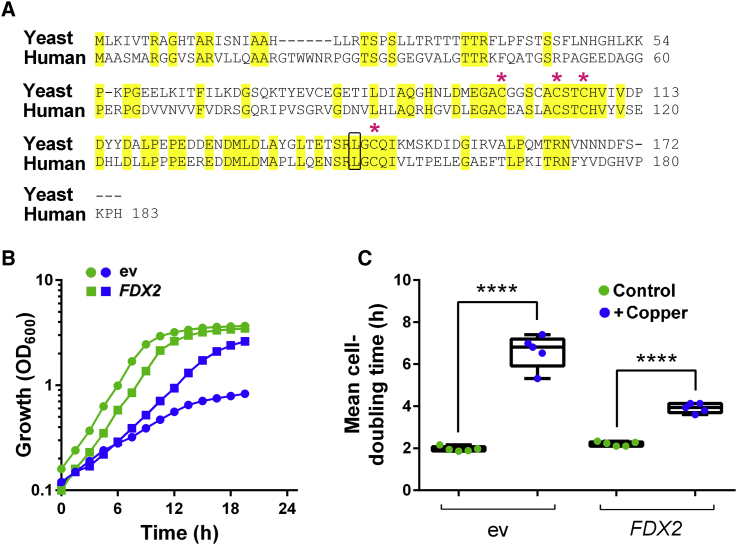
The Human Ferredoxin, Fdx2, Confers Copper Resistance (A) Sequence alignment of human Fdx2 and Yah1 from *S. cerevisiae*. Conserved residues are highlighted in yellow. Asterisks show cysteines involved in the FeS-cluster binding. Leu-142 is framed in black. (B and C) *S. cerevisiae* BY4741 transformed with high-copy *tet* bearing plasmid, empty (ev) or overexpressing human *FDX2* (with the mitochondrial targeting sequence from yeast *SOD2*), was cultured in YNB supplemented (blue) or not (green) with 1.1 mM Cu(NO_3_)_2_. Doxycycline was excluded for maximal *FDX2* expression. (B) One representative set of growth plots (from five independent determinations) is shown. (C) Data from all five experiments are summarized in box plots, with bars indicating the highest and lowest values obtained. Mean cell doubling times were calculated from growth during exponential phase. ****p < 0.0001 according to Student's t test, two tailed.

Human cells possess two mitochondrial ferredoxins, Fdx1 and Fdx2 (annotated as “Fdx1L”). Fdx1 and Fdx2 are both sequence orthologs of yeast Yah1, but only Fdx2 can complement essential Yah1 activities (Sheftel et al., 2010). Fdx2 has 73% similarity and 50% identity with yeast Yah1, including the Leu-142 residue close to the FeS-cluster cavity (Figure 7A). Like Yah1, Fdx2 is essential for FeS-cluster biogenesis (Shi et al., 2012). To indicate whether Fdx2 can be an important target of Cu toxicity, we overexpressed the human protein in yeast (using a *tet*-*FDX2* construct with the mitochondrial targeting sequence of *SOD2*). *FDX2* overexpression conferred resistance to Cu, decreasing the yeast cell doubling time from ∼7 hr to 4 hr in the presence of 1.1 mM Cu(NO_3_)_2_ (Figure 7B). Therefore, dependency of Cu resistance on yeast Yah1 is functionally conserved in the human ferredoxin.

## Discussion

This work points to the essential, conserved mitochondrial ferredoxin as an important target of Cu toxicity in cells. Toxicity of this essential micronutrient can arise from defects in homeostasis or environmental exposures (Bandmann et al., 2015, Pena et al., 1999, Renwick, 2006). Previously described molecular targets of Cu toxicity include cytochrome *c* biogenesis (Durand et al., 2015), oxidative damage to cell constituents such as membrane lipids (Howlett and Avery, 1997), nucleotide synthesis (Johnson et al., 2015), and FeS-protein integrity or biogenesis (Alhebshi et al., 2012, Brancaccio et al., 2017, Foster et al., 2014, Macomber and Imlay, 2009, Tan et al., 2017). Here, Yah1 proved to be a Cu-sensitive weak point of the FeS-cluster biogenesis/delivery pathway in yeast and, as expected for a key target (Avery, 2011), disabling the protein produced Cu-sensitive phenotypes while overexpression of Yah1 or human Fdx2 conferred resistance. We propose that impairment of Yah1 function explains downstream loss of Rli1 function and associated growth inhibition reported previously (Alhebshi et al., 2012). It was FeS-cluster supply to Rli1, which has essential roles in protein synthesis (Nurenberg and Tampe, 2013), that was defective in Cu-exposed cells (Alhebshi et al., 2012). The activities of some nonessential FeS proteins such as aconitase are also Cu sensitive (Foster et al., 2014, Macomber and Imlay, 2009), but these do not account for growth inhibition. Yah1 is required for normal FeS supply to FeS proteins, including Rli1 (Kispal et al., 2005). Here, Yah1 overexpression restored essential function of Rli1 (in ribosome subunit export) under Cu stress, while expression of Yah1^L142V^ with defective FeS stability impaired Rli1 function. The results reveal Yah1 as a new Cu target, accounting for loss of essential Rli1 activity and associated growth inhibition under conditions of Cu excess.

Despite its important functions in biogenesis and assembly of FeS clusters, mitochondrial ferredoxin is one of the less well studied proteins of this pathway. This may be partly due to the fact this protein was linked only recently to human disease (mitochondrial myopathy), unlike Yfh1 (Friedreich's ataxia) and Isu1 (myopathy), among others (Spiegel et al., 2014). There are of course broader consequences of defective FeS biogenesis. Yah1 is also involved in biosynthesis of respiratory-chain components such as heme A (Barros et al., 2002) and coenzyme Q (Pierrel et al., 2010), essential for respiratory growth (but not respiro-fermentative growth as used here). FeS clusters also occur in several respiratory complexes. In HeLa cells, depletion of human Fdx2 causes a decrease in complex I and IV activities (Sheftel et al., 2010). Decreased activities also of complexes I, II, and III were described in cells with decreased Fdx2 content due to a mutation disrupting the ATG translation-initiation site, which is linked to mitochondrial myopathy (Spiegel et al., 2014), and defects in respiratory chain complex activities are known to be associated with other human disorders (Meunier et al., 2013). Therefore, besides downstream effects on Rli1, effects of Cu on respiratory chain function (Djoko and McEwan, 2013, Durand et al., 2015, Hosseini et al., 2014) could also be partly mediated through its effects on mitochondrial ferredoxin.

The principal genetic background used in this study (BY4741) is the same as used in the previous Rli1 work (Alhebshi et al., 2012) and widely elsewhere, being a core background for construction of yeast genomic resources (Winzeler et al., 1999). The BY4741 background carries a mutation in the mitochondrion-related Hap1 transcription factor (Gaisne et al., 1999) and so may give more sensitive detection of factors targeting mitochondrial functions, as anticipated in this study. Accordingly, whereas Yah1 overexpression readily increased Cu resistance in the BY4741 background, this particular phenotype was not detectable against a Hap1-proficient W303 background (C.V. and S.V.A., unpublished data). We could assign this difference to Hap1, as complementation with wild-type Hap1 suppressed the resistance phenotype in BY4741. Moreover, using target inactivation as a more sensitive approach than overexpression (Avery, 2011), achieved in the present study with both the Yah1-CR5 and Yah1^L142V^ mutants (Figures 3, 5, and 6), we did observe Yah1-dependent Cu resistance also in the W303 background. Therefore, Yah1-dependent Cu resistance is not specific to BY4741, although this background facilitates its detection.

Copper has been reported to bind to solvent-exposed FeS clusters of dehydratase enzymes, displacing Fe (Macomber and Imlay, 2009). The Yah1^L142V^ mutant that we constructed here and proved Cu labile was based on previous observations with cyanobacterial ferredoxins, in which a critical amino acid residue (Val versus Leu) localized in the cavities leading to these proteins' clusters was considered to determine relative oxygen accessibility and the proteins' respective oxygen labilities (Singh et al., 2001). A similar cavity occurs in Yah1 (Webert et al., 2014). As well as Cu, the Yah1^L142V^ mutation sensitized cells to the pro-oxidant paraquat (Figure S6) and appeared to increase the lability of the Yah1 FeS cluster even without added Cu, consistent with increased oxygen access. Elevated Fe turnover was accentuated in the presence of Cu, suggesting increased access also of Cu. After reaching an FeS cluster, Cu can bind the sulfur atoms and thereby displace the catalytic Fe atoms (Macomber and Imlay, 2009). This mechanism (versus FeS turnover via Cu-catalyzed ROS formation) is consistent with the present results, particularly considering the discussion below.

ROS such as hydrogen peroxide or superoxide can damage FeS clusters through targeting the catalytic Fe atoms in the proteins (Flint et al., 1993, Jang and Imlay, 2007). Here, in marked contrast to Cu, *YAH1* overexpression caused hypersensitivity to the superoxide-generating agent paraquat (Figure S6). Two considerations may explain these differing outcomes for Cu and paraquat. First, Cu can degrade FeS clusters more extensively than ROS *in vivo* (Macomber and Imlay, 2009). Therefore, should Yah1 retain partial function during ROS action, as does Rli1 in its [3Fe-4S]^+^ state (Barthelme et al., 2007), further degradation by Cu should result in greater Yah1 dysfunction with associated retardation of cell growth, restorable by *YAH1* overexpression. Second, iron released from FeS clusters can participate in Fenton chemistry to aggravate oxidative damage. Excess superoxide (e.g., paraquat generated) should exacerbate these effects of Fe released from overexpressed Yah1 or other FeS protein (Keyer and Imlay, 1996, Liochev and Fridovich, 1999). In contrast, Cu and Fe may primarily be replacing each other. The suggestion that the level of introduced superoxide may determine the different outcomes for paraquat and Cu is supported by the reversal of Yah1 protection against Cu toxicity seen in a *sod2Δ* mutant (Figure 4). This also showed that despite the potential for decreased Sod2 function through Cu mismetallation (Culotta et al., 2006), sufficient Sod2 activity is evidently retained in Cu-exposed wild-type cells to establish the different phenotypes versus *sod2Δ* cells. Regarding maintenance of FeS supply (from Yah1 through to Rli1) under oxidative conditions, its importance is underscored by the report of two non-FeS proteins, Yae1 and Lto1, which facilitate FeS assembly specifically on Rli1 (Paul et al., 2015); these proteins are essential only during aerobic growth.

Our construction of the Yah1^L142V^ mutant with its unstable FeS cluster should prove a useful tool beyond this study. A mutant with increased FeS stability could also be very useful, but increasing the stability of a naturally evolved FeS cluster is a challenging task. We did attempt two approaches to obtain a Cu-resistant Yah1, but neither specific substitution of oxidation-sensitive amino acids near the FeS cluster (e.g., M129L) (Figure S7) nor random mutagenesis of *YAH1* followed by selection for resistant transformants (not shown) successfully produced increased Cu resistance. Perhaps the FeS cluster is already as stable as it can be without adversely affecting Yah1 function. This would not be unexpected from an evolutionary perspective (Imlay, 2006).

What is it about Yah1 that appears to make its Fe(S) content especially unstable to Cu stress? Subcellular localization to the mitochondrion could explain differences versus some of the cytosolic FeS proteins we tested *in vivo*, but similar differences between the test proteins persisted during *in vitro* assays of ^55^FeS turnover (Figure 2A). Solvent exposure of FeS clusters near the protein surface is a common explanation for the ROS labilities of dehydratases (Flint et al., 1993, Liochev and Fridovich, 1999), and a similar relationship was proposed for these enzymes' Cu sensitivities (Macomber and Imlay, 2009). The FeS clusters of Rli1 are predicted to be shielded from solvent (Karcher et al., 2008) and are also resistant to Cu damage (Alhebshi et al., 2012). In contrast, HemN, a bacterial coproporphyrinogen III oxidase involved in heme biosynthesis under anaerobic conditions, has a buried cluster but is susceptible to damage by Cu (Azzouzi et al., 2013, Djoko and McEwan, 2013). The explanation in this case is the presence of a large cleft, which allows coproporphyrinogen III to access the active site and may also allow Cu to enter (Azzouzi et al., 2013). Similarly, the cysteines involved in binding the FeS cluster of Yah1 are buried in the protein, although a cavity that may give Cu direct access to the cluster is present (Webert et al., 2014).

## Significance

**The results show that the FeS content of the essential protein Yah1 is Cu labile and that this can account for associated downstream growth-inhibitory effects of Cu in cells. The ferredoxin that is the focus of this investigation is well conserved in bacteria and eukaryotic mitochondria. We successfully inferred structure**/**function relationships from comparison of the yeast and cyanobacterial ferredoxins in this study. Furthermore**, **the mature form of the human ferredoxin Fdx2 has 50% identity and 73% similarity with the yeast protein. Fdx2 complements Yah1 functionally including**, **we showed**, **in determining the Cu resistance of cells. The other mitochondrial ferredoxin of humans**, **Fdx1**, **is not a functional ortholog of Fdx2 or Yah1** (Sheftel et al., 2010) **but nonetheless also has an important role in FeS-cluster biogenesis** (Shi et al., 2012), **so we cannot discount that it may also contribute to Cu resistance. As discussed in this article**, **the new knowledge generated here could have implications for therapy of Cu-related diseases. It also shows how vulnerability of a specific cellular function can determine when plentiful supply of an essential micronutrient becomes excessive.**

## STAR★Methods

### Key Resources Table

**Table:** 

REAGENT or RESOURCE	SOURCE	IDENTIFIER
**Antibodies**		

Mouse HA tag monoclonal antibody	ThermoFisher Scientific	Cat#26183; RRID:AB_10978021
Goat anti-mouse IgG (H+L) poly-HRP polyclonal secondary antibody	ThermoFisher Scientific	Cat#32230; RRID:AB_1965958
anti-HA beads	Sigma-Aldrich	A2095

**Bacterial and Virus Strains**		

*Escherichia coli* XL1-blue competent cells	ThermoFisher Scientific	Cat#50-125-058
*E. coli* NEB® 5-alpha competent cells	New England Biolabs	Cat#C2987

**Chemicals**, **Peptides**, **and Recombinant Proteins**		

Phusion DNA polymerase	New England BioLabs	Cat#M0530L
hygromycin B	PanReac AppliChem	Cat#A2175,0005
Yeast Nitrogen Base without amino acids	Formedium	Cat#CYN0402
YPD (yeast extract peptone + 2% glucose)	Avery lab	N/A
^55^FeCl_3_	Perkin-Elmer	Cat#NEZ043001MC
Amplification Grade DNase I	Sigma-Aldrich	Cat# AMPD1-1KT
Dihydroethidium	Sigma-Aldrich	Cat#37291
MitoSOX™ Red	Thermo Fisher Scientific	Cat#M36008

**Critical Commercial Assays**		

Q5 Site-Directed Mutagenesis Kit	New England BioLabs	Cat#E0552S
Bradford assay kit	Bio-Rad	Cat#500-0006
Electrochemiluminescence HRP kit	Thermo Fisher Scientific	Cat#32209

**Experimental Models**: **Organisms**/**Strains**		

*Saccharomyces cerevisiae* BY4741 and isogenic single-gene deletion strains	Euroscarf, Frankfurt	N/A
*S. cerevisiae* W303	Barros et al. (2002)	
*S. cerevisiae mxr1Δ*/*mxr2Δ*	Kryukov et al. (2002)	N/A
*S. cerevisiae RLI1*^*C58A*^	Alhebshi et al. (2012)	N/A
*S. cerevisiae* W303 *yah1*::*URA3*	Barros et al. (2002)	N/A
*S. cerevisiae* W303 *yah1*::*hphNT1*	Avery lab.	N/A

**Recombinant DNA**		

pYAH/ST1	Barros et al. (2002)	N/A
pYAH/CR5	Barros et al. (2002)	N/A
pCM190-*YAH1-HA*	Avery lab.	N/A
pRS315-*YAH1*	Avery lab.	N/A
pRS315-*YAH1-HA*	Avery lab.	N/A
pRS315-*YAH1*^*L142V*^*-HA*	Avery lab.	N/A
pRS315-*YAH1*^*M129V*^*-HA*	Avery lab.	N/A
pCM190-*RLI1-HA*	Avery lab.	N/A
pCM190-*NAR1-HA*	Avery lab.	N/A
pCM190-*CFD1-HA*	Avery lab.	N/A
pCM190-*ISU1-HA*	Avery lab.	N/A
pCM190-*YFH1-HA*	Avery lab.	N/A
pCM190-*YAH1-HA*	Avery lab.	N/A
pCM190-*YAH1*^*L142V*^*-HA*	Avery lab.	N/A
pCM190-h*FDX2-MTS*^*SOD1*^*-HA*	Avery lab.	N/A
pCMV-SPORT6-*FDX2*	Dharmacon	Cat#MHS6278-202800851
pCM*HIS*	Avery lab.	N/A
pCM*HIS-YAH1-HA*	Avery lab.	N/A

### Contact for Reagent and Resource Sharing

Further information and requests for resources and reagents should be directed to and will be fulfilled by the Lead Contact, Simon Avery (Simon.Avery@nottingham.ac.uk).

### Experimental Model and Subject Details

#### Yeast Strains and Growth Conditions

*Saccharomyces cerevisiae* BY4741 (*MAT*a; *his3-1*; *leu2-0*; *met15-0*; *ura3-0*) was the principal strain background used throughout the work, and from which isogenic strains were derived as detailed below. *S. cerevisiae* W303 (*MAT*a; *ade2-1*; *his3-1*; *leu2-3*; *112trp1-1*; *ura3-1*; *yah1*::*URA3*) was used only for expression of the *yah1*^*ts*^ allele (CR5). Yeast strains were routinely cultured at 30 C with rotary aeration in YPD broth (Khozoie et al., 2009) or in YNB broth [0.69% yeast-nitrogen base without amino acids (Formedium), 2% (w/v) D-glucose], supplemented as required for plasmid selection with amino acids, adenine or uracil. Where necessary, media were solidified with 2% (w/v) agar (Sigma-Aldrich, St. Louis, MO).

### Method Details

#### Strains and Plasmids

*Saccharomyces cerevisiae* BY4741 (*MAT*a; *his3-1*; *leu2-0*; *met15-0*; *ura3-0*) and the isogenic *sod2Δ*, *isu1Δ*, *ssq1Δ* and *ccc2Δ* strains were from Euroscarf (Frankfurt, Germany). An *mxr1Δ*/*mxr2Δ* (*msra*/*bΔ*) isogenic with BY4741 was constructed previously (*mxr1*::*URA3*; *mxr2*::*KanMX4*) (Kryukov et al., 2002). A *yah1Δ* mutant in the W303 background (*MAT*a; *ade2-1*; *his3-1*; *leu2-3*; *112trp1-1*; *ura3-1*; *yah1*::*URA3*) transformed with single copy plasmids expressing either wild-type *YAH1* (*LEU2* marker) or a *yah1*^*ts*^ allele (CR5; *TRP1* marker) were kind gifts from A. Tzagoloff (Barros et al., 2002). The *RLI1*^*C58A*^ mutant was constructed previously (isogenic with BY4741; *MAT*a; *leu2-0*; *met15-0*; *ura3-0*; *RLI1*^*C58A*^::*HIS3*) (Alhebshi et al., 2012).

To construct mutant versions of *YAH1*, a fragment encompassing the *YAH1* open reading frame (ORF), together with native promoter (500 bp) and terminator (300 bp), was amplified from yeast genomic DNA and ligated between the *Hind*III-*Bam*HI sites of pRS315 (see below). A C-terminal HA tag was added by site-directed mutagenesis using the Q5 Site-Directed Mutagenesis Kit (New England BioLabs) in conjunction with Phusion DNA polymerase. The Leu-142 or Met-129 codons were replaced with a valine codon by site-directed mutagenesis using the Q5 kit with pRS315-*YAH1-HA* as the PCR template. The *YAH1*^*L142V*^ *and YAH1*^*M129V*^ bearing fragments were cloned into pRS315 between the *Hind*III and *Bam*HI sites. To express these constructs in cells, first the *URA3* marker of the *yah1Δ* mutant (complemented with plasmid borne *YAH1*) was replaced by *hphNT1*, with transformants selected on YPD agar supplemented with 150 μg ml^-1^ hygromycin B (PanReac AppliChem). This new strain was transformed with pCM190-*YAH1-HA* (see below) and the original *YAH1*-bearing plasmid removed by repeated subcloning on leucine-containing YNB medium. The resultant strain was transformed with pRS315-*YAH1-HA*, pRS315-*YAH1*^*L142V*^*-HA* or pRS315-*YAH1*^*M129V*^*-HA*, and pCM190-*YAH1-HA* was removed by plasmid shuffling on 5-fluoroorotic acid. A random mutagenesis was also performed, using the PCR technique (Cadwell and Joyce, 1992). The resultant library was used to transform the *yah1Δ* mutant containing pRS315-*YAH1* followed by a selection of any resistant transformants on agar supplemented with Cu(NO_3_)_2_.

For overexpression of FeS proteins, the ORFs *NAR1*, *CFD1*, *ISU1*, *YFH1*, *YAH1*, *YAH1*^*L142V*^ or *FDX2* (with added mitochondrial targeting sequence (MTS) from *SOD2* (Elliott and Volkert, 2004)) were placed under the control of the *tetO* promoter in the pCM190 vector and C-terminally tagged with the HA epitope, as described previously for pCM190-*RLI1-HA* (Alhebshi et al., 2012). *FDX2* was amplified from human *FDX2* cDNA cloned in pCMV-SPORT6 (Dharmacon). ORFs were ligated between the *Not*I-*Pst*I sites (for *CFD1*, *ISU1*, *YFH1*, *YAH1*), *Not*I-*Sbf*I sites (*FDX2*, *YAH1*^*L142V*^) or *Bam*HI-*Pst*I sites (*NAR1*) of pCM190. A pCM*HIS* vector was constructed by inserting the *HIS3* marker between the *Sfo*I and *Eco*RI sites of pCM190. *YAH1-HA* was inserted in the new plasmid between the *Not*I and *Sbf*I sites. This plasmid and the pCM*HIS* empty-vector were used to transform the *mxr1Δ*/*mxr2Δ* strain. All DNA cloning and genetic manipulations were in *Escherichia coli* XL1-Blue cells (Invitrogen) or high-efficiency NEB 5-alpha competent *E. coli* for mutagenesis (New England BioLabs). Yeast transformations were by the lithium acetate method (Gietz and Woods, 2002).

#### Yeast Culturing and Toxicity Assays

Experimental *S. cerevisiae* cultures were inoculated from overnight starter broth-cultures grown from single colonies, and cultured to exponential phase (OD_600_ ∼2.0) in YNB broth medium at 30°C, 120 rev min^-1^. Culture samples were diluted to OD_600_ ∼0.1, and 300-μl aliquots transferred to 48-well plates (Greiner Bio-One, Monroe, NC) before addition or not of Cu(NO_3_)_2_ or paraquat (Sigma-Aldrich, St. Louis, MO). Cultures were incubated with shaking in a BioTek Powerwave XS microplate spectrophotometer, as described previously (Khozoie et al., 2009).

#### ^55^Fe-Labelling and Turnover

For *in vitro* analysis of iron turnover, 200 ml culture that had been pre-incubated for 3 h with ^55^FeCl_3_ (180 μCi L^-1^) (from Perkin-Elmer) was harvested by centrifugation (1500 x *g*, 5 min). Cells were washed and resuspended in lysis buffer (400 μl oxygen-free 50mM phosphate buffer, pH 7.4, 3% (v/v) glycerol, 5mM PMSF (Sigma-Aldrich, St. Louis, MO), EDTA-free protease inhibitor cocktail (Roche, Indianapolis, IN)) together with 500 μl of glass beads, diameter 425-600 μm. Samples were vortexed at maximum speed three times for 1 min, interspersed with three 1-min cooling periods on ice, before centrifugation at 16,000 x *g* for 5 min. Protein in the supernatant was determined with a Bradford assay kit (Bio-Rad, Hercules, CA). Protein (∼500 μl) was mixed with 80 μl anti-HA beads (A2095; Sigma-Aldrich) for 1 h at 4°C. Beads were washed four times with lysis buffer. Aliquots of the beads were incubated aerobically for 10 min at room temperature with 350 μM sodium L-ascorbate and 100 μM histidine (Alhebshi et al., 2012, Macomber and Imlay, 2009) in the absence or presence of Cu(NO_3_)_2_. Beads were collected by centrifugation, suspended in 5 ml scintillation fluid (Emulsifier Safe; Perkin Elmer-Cetus, Waltham, MA), and bead-associated ^55^Fe measured with a Packard Tri-Carb 2100TR liquid scintillation analyzer (Meriden, CT). The measurements of iron turnover from the test proteins *in vivo* were performed exactly as described previously (Alhebshi et al., 2012).

#### Assay of Nuclear Rps2-eGFP Export

Cells transformed with plasmid pRS315-*RPS2-eGFP* (*LEU2* marker) or pRS316-*RPS2-eGFP* (*URA3* marker) (kindly donated by E. Hurt, University of Heidelberg) were examined for cytosolic and/or nuclear localization of fluorescence, as described previously. Cells were viewed with a GX L3201-LED microscope.

#### Western Blotting

For Western blotting, proteins were separated by electrophoresis on 10% (w/v) NuPAGE Bis-Tris gels (Life Technologies) before transfer to nitrocellulose membrane (GE Healthcare). Immunodetection of Yah1-HA was with a mouse anti-HA primary antibody (1:5000 dilution; Thermo Scientific) and poly horseradish peroxidase (poly HRP)-conjugated goat anti-mouse antibody (1:5000 dilution; Thermo Scientific). Yah1-HA was detected with an electrochemiluminescence HRP kit (Pierce) and imaged using a Chemidoc XRS (Bio-Rad). Protein-band intensities were quantified with ImageJ software.

#### RNA Extraction and Quantitative RT-PCR (qRT-PCR)

mRNA from specified genes was quantified by qRT-PCR exactly as described previously (Halliwell et al., 2012), except that RNA was isolated by the “hot phenol” technique then treated with Amplification Grade DNase I (Sigma-Aldrich, St. Louis, MO), and 25 ng cDNA with 175 nM gene*-*specific primers (sequences available on request) were used in the PCR reactions. PCRs were carried out for 40 cycles; denaturation at 95°C for 15 s, annealing/extension at 60°C for 30 s. Melting-curve analysis confirmed a single PCR product. Amplification was quantified from a standard curve constructed from reactions with defined genomic DNA concentrations.

#### ROS Accumulation

ROS accumulation in cells was determined with the fluorescent probe dihydroethidium (DHE) (Giorgini et al., 2005) or for mitochondria-specific assay with MitoSOX™ Red (Thermo Fisher Scientific). Samples of yeast culture (1.5 ml at OD_600_∼2.0) were centrifuged, washed and then incubated in 100 μl PBS, 5 μM DHE or MitoSOX for 30 min at 30°C, 120 rev min^-1^. Cells were harvested by centrifugation and resuspended in 500 μl PBS before analysis of cellular DHE or MitoSOX fluorescence with a Beckman Coulter FC500 cytometer equipped with a 488 nm laser. Emitted fluorescence was collected with either 625/26 (DHE) or 575/25 (MitoSOX) band pass filters.

### Quantification and Statistical Analysis

All tests for significance were according to Student’s *t*-test, two tailed, by comparison with the relevant control as specified in the figures or figure legends. At least three independent experiments were performed in each case (n ≥3), as detailed in the relevant figure legends. Significance was defined by p values determined from the *t*-tests, and indicated on figures as *, p<0.05; **, p<0.01; ***, p<0.001; ****, p<0.001.

## Author Contributions

C.V. and S.L.H. performed the experiments. All authors participated in the design of the study, interpretation of data, and writing of the manuscript. S.V.A. conceived the study. All authors read and approved the final manuscript.

## Supplemental Information

Document S1. Figures S1–S7

Document S2. Article plus Supplemental Information
