# Supplementary material for: Mitochondrial Ferredoxin Determines Vulnerability of Cells to Copper Excess
Source: Cell Chem Biol. 2017 Oct 19;24(10):1228–1237.e3. doi: 10.1016/j.chembiol.2017.08.005 (PMC5654725; doi:10.1016/j.chembiol.2017.08.005)
Supplement: Document S1. Figures S1–S7 [file mmc1.pdf]

Cell Chemical Biology, Volume *24*

Supplemental Information

Mitochondrial Ferredoxin Determines Vulnerability of Cells to Copper Excess

Cindy Vallières, Sara L. Holland, and Simon V. Avery

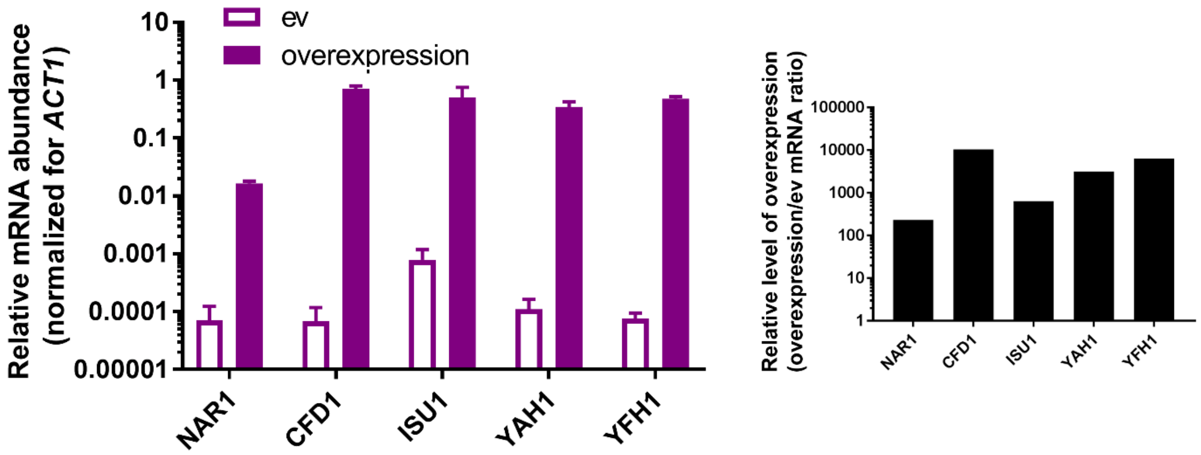

Figure S1. Related to Figure 1. qRT-PCR analysis of overexpression. RNA was extracted from wild type cells transformed with pCM190 (+ev) or one of the pCM190-*tetORF* constructs (the relevant ORF is indicated on the figure) after overnight growth in YNB medium (without doxycycline). mRNA corresponding to the different ORFs was analysed with qRT-PCR, performed in triplicate for each condition.

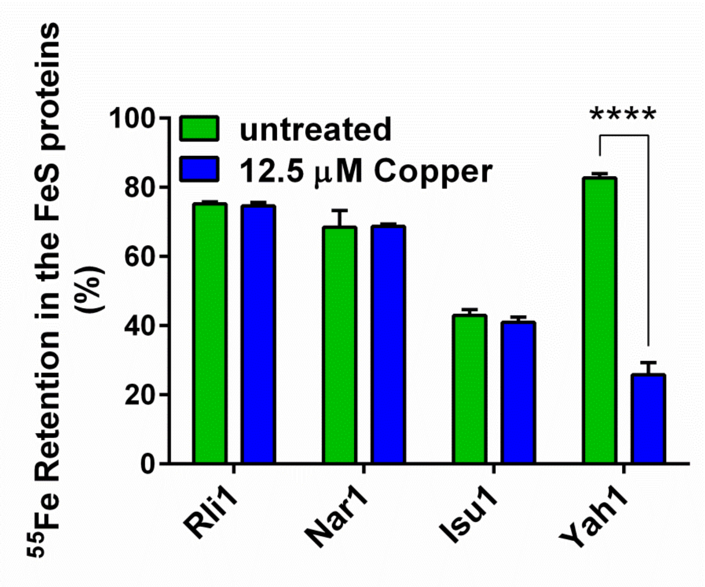

Figure S2. Related to Figure 2. ^{55}Fe turnover during *in vitro* copper treatment of key proteins involved in FeS-cluster biosynthesis/delivery. Yeast expressing HA-tagged constructs of the specified proteins, under *tet* control from high-copy plasmids, were cultured in the absence of doxycycline to maximize expression [giving yields of between 0.6 (Isu1-HA) and 1.6 (Yah1-HA) x 10^6 cpm (g cells)$^{-1}$ in immunoprecipitates of the proteins after labelling as follows]: HA-tagged, ^{55}Fe-labelled proteins were immunoprecipitated from protein extracts of cells preincubated with $^{55}FeCl_3$. ^{55}Fe retention in the FeS proteins was measured after subsequent 10 min incubations with 350µM ascorbate/100µM histidine supplemented or not with 12.5µM $Cu(NO_3)_2$. ****, $p<0.0001$ according to Student's *t*-test, two tailed. All values are means from at least three independent experiments ± SEM.

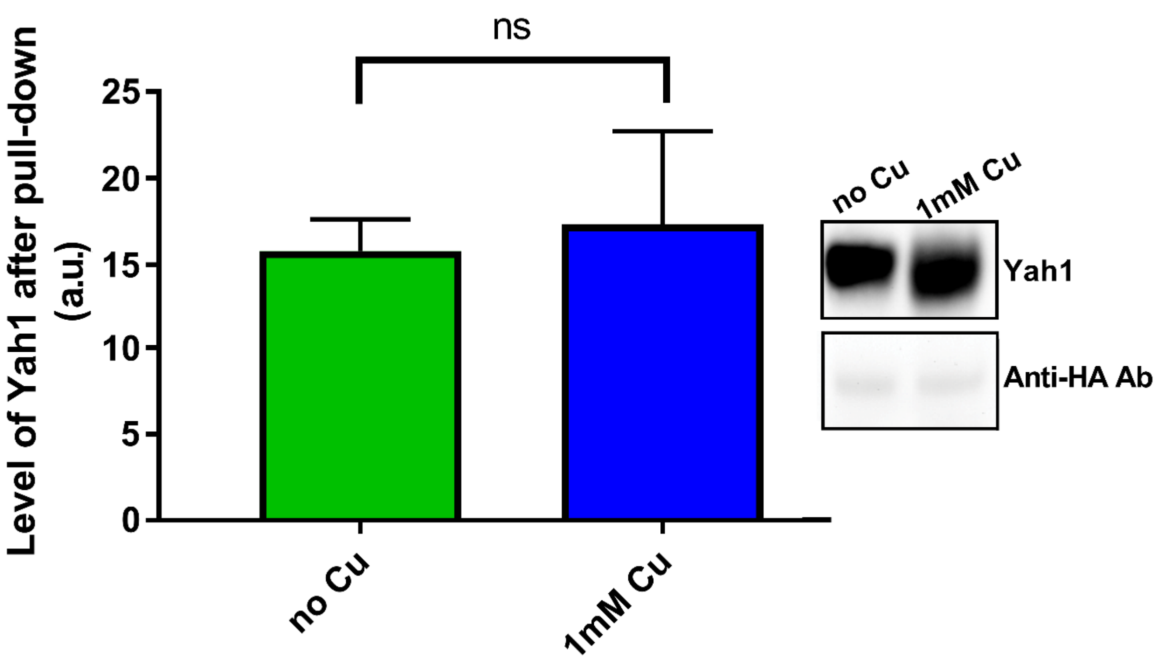

Figure S3. Related to Figure 2. Western analysis of Yah1 pulled down from cultures treated or not with copper. Yeast expressing Yah1-HA under *tet* control were cultured in the absence of doxycycline to maximize expression. Cells were incubated for 1 h in the absence or presence of 1 mM $Cu(NO_3)_2$ before immunoprecipitation of Yah1-HA and analysis by western blot and densitometry. The level of Yah1 was normalized for the anti-HA antibody used for the pull down. Representative western blots are shown, with means ± SEM from quantitation of three independent experiments. a.u., arbitrary units; ns, not significant.

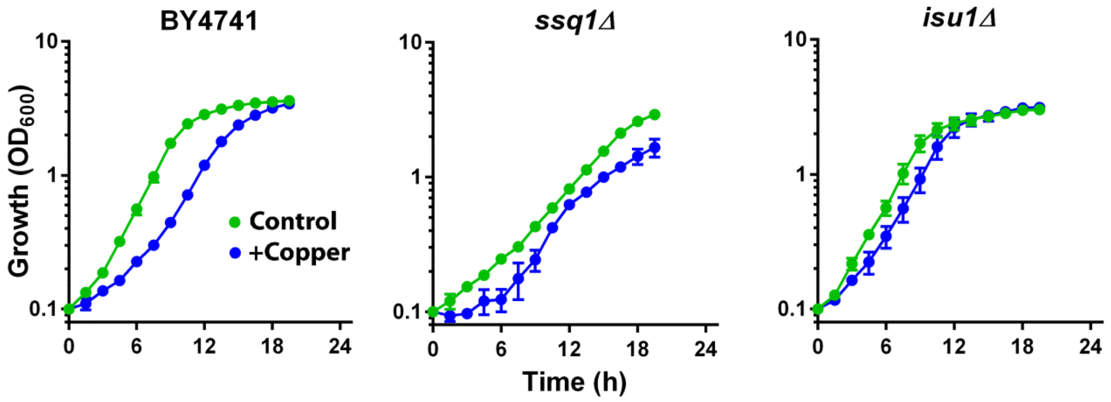

Figure S4. Related to Figure 3. Lack of copper-sensitization in iron hyper-accumulation mutants. Wild type cells and isogenic *isu1Δ* or *ssq1Δ* mutants (known to hyper-accumulate Fe) were cultured in YNB supplemented or not with 0.7 mM $Cu(NO_3)_2$. Mean data are shown from triplicate independent growth experiments ± SEM.

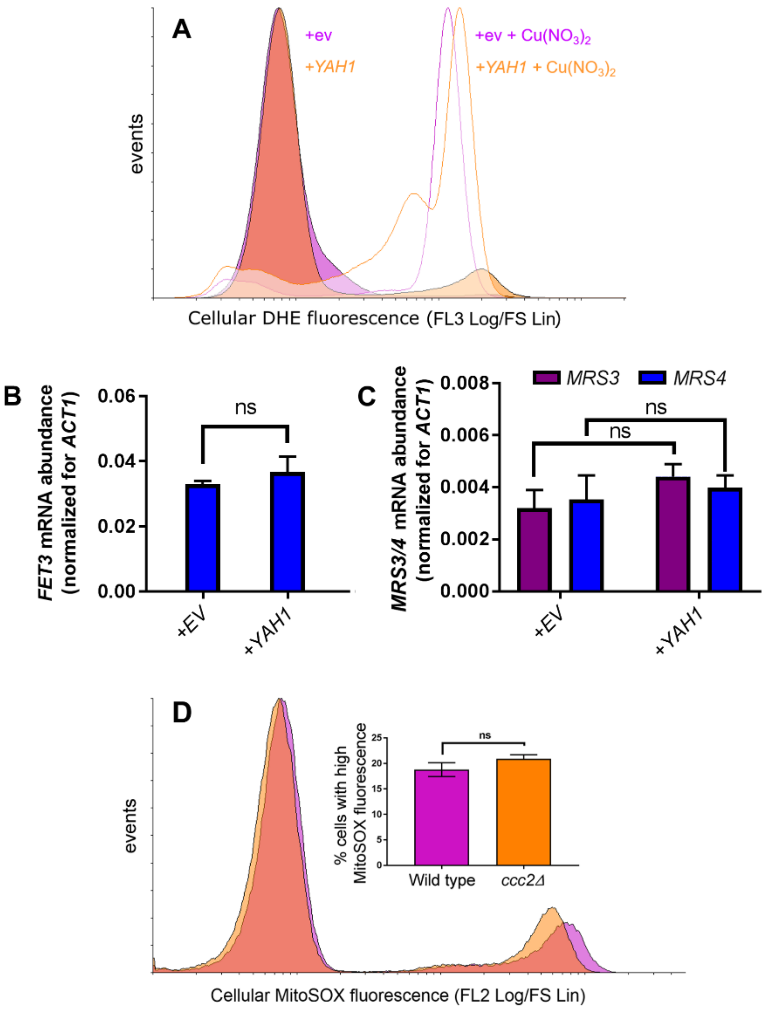

Figure S5. Related to Figure 3. The level of Yah1 or Ccc2 expression has little effect on ROS measurements and *FET3, MSR3 or MSR4* expression are unaffected by Yah1 overexpression. Wild type cells transformed with high-copy *tet* bearing plasmid, empty ("+ev") or overexpressing *YAH1* ("*+YAH1"*) were cultured in YNB medium. (**A**) After 4 h growth in the absence or presence of 0.9 mM $Cu(NO_3)_2$, cells were stained with the ROS probe dihydroethidium (DHE) before analysis by flow cytometry. DHE fluorescence collected in the FL3 channel was corrected for cell volume (FS, forward scatter). Each histogram shows data collected for 100,000 cells. (**B,C**) After overnight incubation with 0.6 mM $Cu(NO_3)_2$, RNA was extracted and *FET3* and *ACT1* (B) or *MRS3*, *MRS4* and *ACT1* (C) mRNA levels analysed in triplicate with qRT-PCR; ns, not significant. (**D**) Exponential cultures of wild type or *ccc2Δ* cells were stained with MitoSOX and analysed by flow cytometry. The proportions of cells in the high-MitoSOX (high mitochondrial ROS) sub-population were determined after gating. ns, no significant difference.

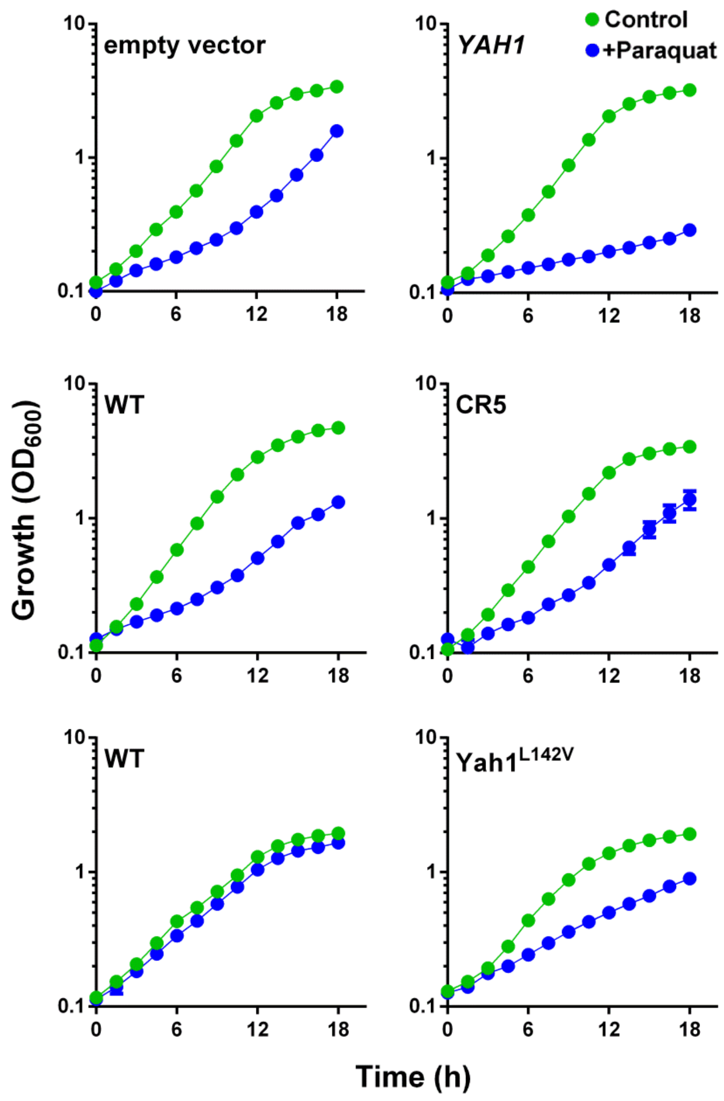

Figure S6. Related to Figure 5. Influence of Yah1 on cellular paraquat sensitivity (see Discussion). Top row: Wild type cells transformed with high-copy *tet* bearing plasmid, empty or overexpressing *YAH1* ("*YAH1*") were cultured in YNB medium supplemented or not with 0.7 mM paraquat. Middle row: *yah1Δ ::URA3* cells transformed with single copy plasmids to express either the wild type *YAH1* (WT) or the CR5 mutant version were cultured in YNB supplemented or not with 0.7mM paraquat. Last row: *yah1Δ* cells transformed with single copy plasmids to express either the wild type *YAH1* (WT) or the Yah1^{L142V} mutant version were cultured in YNB supplemented or not with 0.2mM paraquat. Mean data are shown from triplicate independent growth experiments ± SEM.

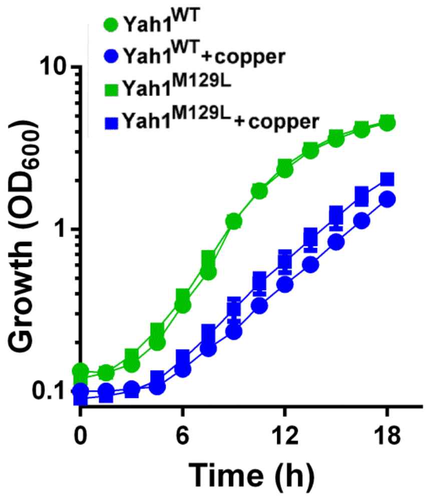

Figure S7. Related to Figure 5. Expression of a Yah1^{M129L} mutant does not alter copper sensitivity (see Discussion). *yah1Δ* cells expressing either the wild type *YAH1* or *YAH1^{M129L}* genes from single copy plasmids were cultured in YNB medium supplemented or not with 0.5 mM $Cu(NO_3)_2$. Mean data are shown from triplicate independent growth experiments ± SEM.
